# Evolvable AI needs operational risk thresholds

**DOI:** 10.1073/pnas.2618781123

**Published:** 2026-07-10

**Authors:** Shweta Rana, Harpreet Singh

**Affiliations:** ^a^ Division of Development Research, Indian Council of Medical Research, New Delhi 110029, India; ^b^ Faculty of Medical Sciences, Academy of Scientific and Innovative Research, Ghaziabad 201002, India

Müller, Steels, and Szathmáry made this timely case that evolvable AI (eAI) should be treated as a distinct class of risk because essential elements of life such as replication, heredity, variation, and selection were also created in digital systems (1). Their distinction between human-controlled “breeder” settings and open “ecosystem” settings is especially useful. However, this distinction will remain difficult to govern unless it is translated into measurable thresholds. The policy consequence of this distinction also remains underspecified. Before regulators can control replication, control heredity, or reshape selection, they need a reproducible framework to determine when an AI deployment has acquired enough evolvability to warrant such controls.

We suggest that eAI governance should include an explicit “evolvability risk assessment” to existing AI risk-management practice. Such an assessment should not ask only whether a model is capable, biased, deceptive, or dual-use. It should ask whether the surrounding agentic system contains a functional evolutionary loop. Recent work on Continual Harness illustrates why such assessment is becoming urgent: An embodied foundation-model agent can refine its prompt, subagents, skills, and memory online during a continuous run, and can couple harness refinement with model updating without resetting the environment (2). At minimum, this requires assessment of five properties: autonomous replication or instance creation; heritable persistence of prompts, memories, adapters, tools, code, or policies; mechanisms for variation through rewriting, fine-tuning, merging, tool creation, or self-modification; selection pressures arising from rewards, benchmarks, engagement, market performance, survival, or evasion; and environmental access through browsers, shells, APIs, cloud resources, robots, or institutional workflows.

This distinction matters because many current AI governance frameworks focus primarily on intended use, model capability, transparency, human oversight, cybersecurity, and postmarket monitoring (3, 4). These remain necessary, but they do not by themselves identify the transition from a static or episodically updated AI system to a population of persistent, modifiable, and selectable agents. Similarly, deception testing is necessary (5), but deception becomes more consequential if deceptive variants can persist, recombine, and be selected across deployments.

The authors rightly argue that adapters, fine-tunes, and merges should be treated as genetic material (1). That insight can be made operational. Developers of high-impact agentic systems should be required to maintain lineage records for system prompts, memory states, tool libraries, adapters, model merges, reward models, and deployment configurations. Release decisions should then be linked to explicit thresholds: no autonomous cloud acquisition; no unsupervised self-deployment; signed and revocable agent variants; sandboxed execution; independent logging of high-risk tool calls; and prespecified shutdown tests that verify corrigibility under distribution shift.

The central contribution of the eAI framing is that it shifts the safety question from “How capable is this model?” to “Can this system become a lineage?” This should now be translated into audit standards. Without operational thresholds, the warning may remain persuasive but difficult to enforce.

## Data Availability

There are no data underlying this work.
